# Acute Kidney Injury Recovery Patterns in ST-Segment Elevation Myocardial Infarction Patients

**DOI:** 10.3390/jcm11082169

**Published:** 2022-04-13

**Authors:** Tamar Itach, Ariel Banai, Yael Paran, David Zahler, Ilan Merdler, David Eliashiv, Shmuel Banai, Yacov Shacham

**Affiliations:** 1 Departments of Cardiology, Tel-Aviv Sourasky Medical Center Affiliated to the Sackler Faculty of Medicine, Tel-Aviv University, Tel-Aviv 6423906, Israel; itachtamar@gmail.com (T.I.); arielbanai@gmail.com (A.B.); david.zahler@gmail.com (D.Z.); ilanmerdler@gmail.com (I.M.); deliashiv@gmail.com (D.E.); shmuelb@tlvmc.gov.il (S.B.); 2 Departments of Internal Medicine, Tel-Aviv Sourasky Medical Center Affiliated to the Sackler Faculty of Medicine, Tel-Aviv University, Tel-Aviv 6423906, Israel; yaelparan1@gmail.com

**Keywords:** acute kidney injury, ST-segment elevation myocardial infarction, percutaneous coronary intervention, cardiac intensive care unit

## Abstract

Background: Acute kidney injury (AKI) is a frequent complication in patients with ST-segment elevation myocardial infarction (STEMI) undergoing percutaneous coronary intervention (PCI). Identification of different AKI recovery patterns may improve patient prognostic stratification. We investigated the clinical relevance of AKI recovery patterns among STEMI patients undergoing PCI. Methods: A retrospective study of 2943 STEMI patients undergoing PCI. The incidence of renal impairment, in-hospital complications, short and long-term mortality, were compared between patients without AKI, with early recovery defined as a return to baseline creatinine within 72 h, and no AKI recovery/delayed recovery defined as all other AKI cases. Results: A total of 255 (8.7%) patients developed AKI, of whom 124/255 (49%) patients had an early recovery, whereas 131/255 (51%) had no AKI recovery/delayed recovery. Patients without recovery were more likely to have in-hospital complications and higher long-term mortality (36.64% vs. 7.25%%; *p* < 0.001). In a multivariable regression model, the mortality hazard ratio (HR) for long term mortality remained significant for patients with no/delayed recovery AKI (HR 7.76, 95% CI 4.69 to 12.86, *p* < 0.001), and a strong trend among patients with resolving AKI (HR 2.09, 95% CI 0.933–4.687, *p* = 0.071). Conclusions: Among STEMI patients undergoing PCI, the recovery pattern of AKI is a valuable prognostic marker.

## 1. Introduction

Acute kidney injury (AKI) is a frequent and serious complication in patients with ST-elevation myocardial infarction (STEMI) treated by percutaneous coronary intervention (PCI) and associated with a prolonged hospital stay, increased morbidity and mortality rates [1,2,3]. Most studies to date focused on the severity of AKI despite increasing recognition that renal recovery following AKI is an important outcome and little data defining this outcome is available [4].

The Acute Dialysis Quality Initiative (ADQI) group recently proposed criteria for renal recovery as the absence of AKI by both serum creatinine (sCr) and urine output criteria within 7 days after AKI onset [5]. A recent study identified AKI reversal patterns and their association with long-term prognosis, with a better prognosis in the early sustained reversal (within 7 d), compare with late reversal (beyond 7 d) and no reversal [6]. Patients who fail to recover had and higher morbidities and mortality [7].

Several studies defined recovery after 3–7 days to make the distinction between transient and persistent AKI and even suggest that the 72-h period after AKI distinguishes the risk of clinically important long-term outcomes [4,8]. Hence, the identification of different AKI recovery patterns may improve patient risk stratification and prognostic elaboration.

Determinants of renal recovery and pattern may be also influenced by the etiology of AKI [9]. We investigated the incidence and possible clinical relevance of AKI recovery patterns among STEMI patients undergoing primary PCI who developed AKI throughout their hospitalization.

## 2. Materials and Methods

### 2.1. Patients

A retrospective, single-center observational study was performed at the Tel-Aviv Sourasky Medical Center, a tertiary referral hospital with a 24/7 primary PCI service. We evaluated 2967 patients admitted between December 2007 and December 2019 to the Cardiac Intensive Care Unit (CICU) with the diagnosis of acute STEMI. Of these patients, we excluded patients requiring chronic peritoneal or hemodialysis (*n* = 5) treatment, as well patients with a missing record of renal function beyond acute insult or discharge (*n* = 19). The final cohort consisted of 2943 patients whose baseline demographic, cardiovascular history, clinical risk factors, treatment characteristics, and laboratory results were retrieved from their hospital electronic medical files.

Diagnosis of STEMI was established by a typical history of chest pain, diagnostic electrocardiographic changes, and serial elevation of serum cardiac biomarkers [10]. Primary PCI was performed in patients with symptoms ≤12 h in duration, and in patients with symptoms lasting 12–24 h if pain consisted at the time of admission. Symptom duration was defined as the time from symptom onset (usually chest pain or discomfort) to ER/catheterization laboratory admission. Following coronary interventional procedures, 0.9% saline was given intravenously at a rate of 1 mL/kg/h for 12 h after contrast exposure. In patients with overt heart failure, the hydration rate was reduced at the discretion of the attending physician. The contrast medium used in procedures was iodixanol (Visipaque, GE healthcare, Ireland) or iohexol (Omnipaque, GE healthcare, Ireland).

Patient records were evaluated for the in-hospital course and occurrence of adverse outcomes. These included the development of respiratory failure with the need for mechanical ventilation, cardiogenic shock with the need for Intra-aortic balloon pump (IABP), new-onset atrial fibrillation (AF), ventricular tachycardia (VT)/fibrillation (VF) episodes, as well as in-hospital mortality. 1-year mortality was assessed following hospital discharge was determined from computerized records of the population registry bureau.

The study was conducted according to the guidelines of the Declaration of Helsinki, and approved by the Tel-Aviv Sourasky medical center Helsinki committee (Institutional Review Board code: TLV-16-0224). Informed consent was obtained from all subjects involved in the study.

### 2.2. Definition of AKI and AKI Recovery

The serum creatinine level was determined upon hospital admission, prior to primary PCI, and at least once a day during the CICU stay and/or step-down until hospital discharge, and was available for all analyzed patients. The estimated glomerular filtration rate (eGFR) was estimated using the Chronic Kidney Disease Epidemiology Collaboration (CKD-EPI) equation [11]. Chronic kidney disease (CKD) was categorized as admission eGFR of <60 mL/ min/1.73 m² 14. AKI was determined using the “kidney disease: improving global outcomes (KDIGO)” criteria [12], and was defined as an increase in serum creatinine ≥0.3 mg/dl within 48 h of admission or an increase in serum creatinine ≥1.5 times baseline, which was known or presumed to have occurred within the prior 7 days. AKI staging was determined according to KDIGO criteria, stage 1 was defined as a 1.5–1.9 times increase in baseline serum creatinine, stage 2 as a 2.0–2.9 times increase and stage 3 as 3.0 times increase or the initiation of renal replacement therapy [12].

Data on urine output throughout hospitalization was not available for patients included, and thus not used to define AKI.

Resolving AKI was defined as a return to baseline creatinine or return to creatinine level within 0.3 mg/dl of that at baseline. Early recovery was defined as resolving AKI within 72 h after AKI diagnosis and no recovery/delayed recovery as all AKI cases not meeting the definition of resolving AKI within 72 h [8].

Based on laboratory data following hospital discharge, long-term renal outcomes were amended during 90 days follow-up after exposure to an AKI event. The most commonly used ADQI criteria define acute kidney disease (AKD) as AKI persisting beyond 7 days. In the present cohort, where recovery was defined at 72 h, patients who returned to baseline creatinine within 90 days were referred as having “modified AKD”, while patients who did not return to baseline were defined as progressing to CKD.

### 2.3. Statistical Analysis

All data were summarized and displayed as mean (±standard deviation) or median (25–75%) for continuous variables unless stated otherwise, and as number (percentage) of patients in each group for categorical variables. The *p*-values for the categorical variables were calculated with the chi-square test. Continuous variables were compared using the independent sample *t*-test or the Mann-Whitney U test. Analysis of variance (ANOVA) test was performed for the comparison between patients with early renal recovery, late recovery and no recovery/delayed recovery. To evaluate whether renal recovery pattern was associated with adverse in-hospital outcomes we used a backward wald logistic regression adjusted for all parameters found significant in the univariate analysis. The Kaplan-Meier method and log-rank test were used to evaluate the association between renal recovery pattern and survival. To assess if renal recovery pattern was associated with 1-year mortality, we used multivariate Cox Regression adjusted for all baseline variables found to be significant in the univariate analysis. A two-tailed *p*-value of < 0.05 was considered significant for all analyses. All analyses were performed with the SPSS software (SPSS Inc., Chicago, IL, USA).

## 3. Results

A total of 2943 patients were included in the study, of whom 255 patients (8.7%) had AKI during hospitalization, whereas 124/255 (49%) patients had an early renal recovery AKI and 131/255 (51%) had a non-resolving AKI. Out of 255 patients with AKI, 189 (73%) had a stage 1 AKI, 38 (15%) had a stage 2 AKI, 19 (7.5%) had a stage 3 AKI and, 5/19 (26%) needed a renal replacement therapy. Demographic and clinical baseline parameters stratified by renal injury and recovery pattern are shown in Table 1. Compared with the non-AKI group, Patients with AKI were significantly older, female gender, higher prevalence of comorbidities, higher eGFR, baseline and peak sCr. As well, patients with AKI had a longer time to the emergency room (ER) and to reperfusion while no significant difference was noted between door to balloon time. In addition, a significant difference in the severity of coronary artery disease (CAD), while patients with AKI had a more severe CAD. Comparing AKI patients, lower median eGFR and a higher baseline and peak sCr were eminent at patients without renal recovery. In addition, they were significantly older, more of them had a family history of IHD and a higher Troponin-I level. Whereas no significant difference in time to ER or reperfusion and in the severity of CAD.

Out of 131 patients with non-resolving AKI, 58 (44%) had a normal renal function before admission, while 73 (56%) had CKD with acute on chronic renal injury.

### 3.1. In-Hospital Outcome

**Figure 1:**
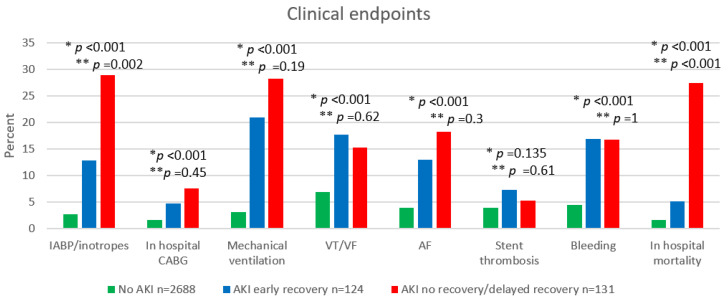
In-hospital adverse outcomes. Abbreviations: AKI, acute kidney injury; IABP, intra-aortic balloon pump; CABG, Coronary artery bypass graft surgery; VT, ventricular tachycardia; VF, ventricular fibrillation; AF, atrial fibrillation. * *p*-value for the comparison between AKI group reversal patterns. ** *p*-value for the comparison between AKI early recovery vs. AKI no recovery/delayed recovery.

Figure 1, presents the key in-hospital adverse outcomes according to AKI group reversal patterns. Overall, patients with any AKI had worse outcomes, but patients with no AKI recovery suffer from a higher rate of in-hospital adverse outcomes, as well as 30-day mortality, Table S1.

In order to elucidate the major risk factors for no-renal recovery following AKI we created a multivariate regression model. The major risk factors were older age (HR 1.063, 95% CI 1.04–1.08, *p* < 0.001), family history of IHD (HR 0.33, 95% CI 0.13–0.85, *p* = 0.02), hypertension (HR 2.83, 95% CI 1.73–4.61, *p* < 0.001), CAD (HR 1.3, 95% CI 1.03–1.65, *p* = 0.02) and a lower ejection fraction (HR 0.92, 95% CI 0.89–0.93, *p* < 0.001), Table 2.

### 3.2. Long Term Mortality

**Figure 2:**
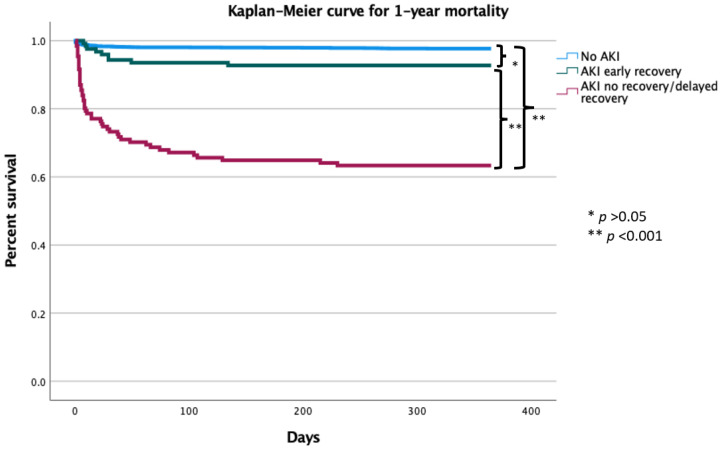
Cumulative survival rates for 2943 patients with STEMI based on AKI resolution. Abbreviations: AKI, acute kidney injury. * *p*-value > 0.05, ** *p*-value < 0.001.

There was a graded increase in 1 year mortality among patients with no AKI, early recovery AKI and non-recovery AKI (2% vs. 7% vs. 37%, *p* < 0.001), Figure 2. Compared to patients with no AKI the hazard ratio for 1-year mortality was 17.425 (95% CI 12.07–25.15; *p* < 0.001) for patients with non-resolving AKI and 1.87 (95% CI 0.947–3.687; *p* = 0.071) for patients with resolving AKI. In a Multivariate cox regression model (Table 3), non-resolving AKI was independently associated with increased risk for 1-year mortality (HR 7.76, 95% CI 4.69 to 12.86, *p* < 0.001), with a strong trend among patients with resolving AKI (HR 2.09, 95% CI 0.933–4.687, *p* = 0.071).

### 3.3. Long Term Renal Outcomes for Non-Recovered AKI

During 90 days follow-up after an AKI event, 63 (48%) patients returned to baseline creatinine, therefore, had modified AKD. While 68 (52%) patients did not return to baseline, therefore had a new-onset CKD or worsened their renal impairment if already suffered from CKD, 5 of whom required the onset of hemodialysis. Among patients with early AKI recovery (*n* = 124), 21 (17%) patients had renal impairment at 3 months after discharge, even though their renal function returned to baseline post-AKI.

## 4. Discussion

This study demonstrated that among STEMI patients treated with primary PCI, the pattern of AKI recovery is a prognostic marker for adverse outcomes and mortality. The main finding in our study is even patients with early resolution of renal function had higher rates of adverse in-hospital outcomes as well, a higher mortality rate. Furthermore, although returning to normal renal function prior to discharge, up to 17% of patients with early AKI resolution are prone to suffer from deterioration of renal function in the future, therefore making them more susceptible to future renal impairment. Additionally, patients with non-resolving AKI had adverse outcomes and a higher 1-year mortality compared to patients with no AKI and early recovery AKI.

It is well established that among STEMI patients treated with PCI, AKI is a risk factor for worse outcome and higher mortality rate [13,14,15]. As well, dynamic changes in renal function during acute myocardial infarction are strongly related to long-term mortality [16]. Data from the National Cardiovascular Data Registry/Cath-PCI registry including patients undergoing PCI suggest that approximately 7% of patients experienced AKI and in patients undergoing primary PCI for acute myocardial infarction, AKI occurs more frequently with rates up to 20% [17]. Nevertheless, only restricted data is available regarding the incidence and clinical relevance of AKI recovery patterns among STEMI patients undergoing primary PCI.

Most studies striving to recognize risk factors for poor outcomes following AKI have focused on the severity of AKI or degree of recovery. An overlooked indicator may comprise the timing of recovery despite increasing recognition that renal recovery after AKI is an important outcome and little data defining this outcome is available [18]. At the time the Acute Dialysis Quality Initiative (ADQI) proposed the Risk, Injury, Failure, Loss of Kidney Function and End-Stage Renal Disease (RIFLE) criteria for AKI, they also proposed criteria for recovery as the absence of AKI by both serum creatinine and urine output criteria (per KDIGO) within 7 days after AKI onset [12,19]. Patients who fail to recover will have a significantly shorter life span and higher morbidities [7].

In 2017, Kellum et al. identified in their study of AKI reversal patterns, five distinct recovery phenotypes: (1) early sustained reversal (within 7 d), (2) late reversal (after 7 d), (3) early reversal with one or more relapses but ultimate recovery, (4) early reversal with one or more relapses and no recovery, and (5) no reversal at all [20]. Characterization of recovery patterns in this study was restricted to hospitalization, and long-term kidney outcomes were not examined. Recent studies demonstrate that complete and sustained reversal of an AKI episode within 48–72 h of its onset is associated with better outcomes than longer durations of AKI [5], as was also demonstrated in our cohort.

Limited studies have focused on the pattern and duration of recovery, especially when extending beyond discharge. In 2012, the AKI study group “kidney disease: improving global outcomes (KDIGO)” proposed the term AKD as the course of disease after AKI, which describes acute or subacute damage and/or loss of kidney function for a duration between 7 and 90 days after exposure to an AKI initiating event, whereas CKD is defined by the persistence of kidney disease for a period of >90 days [5]. In our cohort, more than 50% of patients with non-resolving AKI eventually progressed to CKD whereas the rest had AKD during 90 days follow up after exposure to an AKI. Furthermore, our study demonstrated even patients with early reversal of an AKI had a worse outcome than patients with no AKI and were prone to subsequent renal impairment after initial recovery.

Determinants of renal recovery and pattern may be influenced by the etiology of AKI [9]. In a recent study defining patterns of recovery in critically ill patients with stage 2 or 3 AKI, sepsis-associated AKI was allied with an increased risk of relapse compared with patients with early sustained reversal [6]. In another study, patients after coronary angiography who had AKI were allied with a greater tendency of sustained loss of kidney function beyond 3 months and were more likely to progress toward ESRD. These events were associated with the severity of AKI and the proportion of patients who fail to recover increased with greater severity of AKI [15].

The pathophysiology of AKI in the setting of STEMI is complex, especially in those undergoing primary PCI and goes beyond the administration of contrast media [21,22]. Data on AKI in STEMI patients undergoing PCI shows that even a small elevation (> 0.3 mg/dL) in serum creatinine is associated with a poorer prognosis even after normalization of renal function prior to hospital discharge [14,23]. As noted in our cohort, even patients with AKI resolution before hospital discharge may represent an at-risk population for adverse outcomes, and as shown at 3 months follow up were prone to subsequent renal impairment after initial recovery, hence a strict follow up should be emphasis for patients who had AKI, those with non-resolving AKI and resolved AKI. In addition, these patients are more vulnerable to renal injury which has a clinical and treatment implication, especially regarding medical treatment which may impair their renal function, such as ACE-I/ ARB or during a staged PCI. Likewise, further research should focus on the effects of interventions to prevent renal injury and once occurred to slow progression into CKD in non-resolving AKI.

Our findings bear several important limitations. This was a single-center retrospective and non-randomized observational study; because of its retrospective nature, the study was subjected to selection bias, and therefore the results point toward association, and not cause and effect. Furthermore, although AKI definition refers to a serum creatinine increase compared to the baseline value, the serum creatinine at hospital admission may not represent a true baseline value in STEMI patients as an increase could have already occurred prior to hospital arrival owing to hemodynamic impairment. Criteria to define AKI are based either on changes in sCr or in urine output. As data on urine output throughout hospitalization was not available for patients included, some cases of AKI may have been missed.

In the current definition of AKI baseline sCr, is determined from outpatient values, over 7–365 days prior to hospitalization. The utilization of sCr on admission may lead to an error in AKI determination and the definition of AKI resolution. Labeling patients as CKD based on admission eGFR in the setting of STEMI is erroneous and will label all patients with community acquired AKI as CKD.

As well, data regarding concomitant therapy or other confounders with effect on AKI development throughout hospitalization was not present and their effect on AKI development could not be assessed. Among patients with no AKI, laboratory follow up was not available at 90 days, hence the number of patients in this groups having CKD at 90 days could not been assessed.

## 5. Conclusions

Among STEMI patients after primary PCI, the recovery pattern of an AKI event is a valuable prognosis marker. Non-resolving AKI could be associated with a worse prognosis than early AKI recovery, but even early resolution of renal function allied with adverse in-hospital outcomes, long-term mortality, and prone to relapse. We recommend carefully monitoring STEMI patients with AKI, especially those who failed to return to baseline renal function after 72 h and post-hospital discharge.

## Figures and Tables

**Table 1:** Baseline Characteristics.

	No AKI *n* = 2688	AKI Early Recovery *n* = 124	AKI No Recovery/ Delayed Recovery *n* = 131	No AKI vs. AKI Early Recovery	No AKI vs. AKI No Recovery/ Delayed Recovery	AKI early Recovery vs. AKI No Recovery/ Delayed Recovery	*p*-Value
Age, years	60.9 ± 12.7	67.7 ± 12.2	75.7 ± 12.5	<0.001	<0.001	0.001	<0.001
Male gender	2213 (82.3)	91 (73.4)	95 (72.5)	0.016	0.007	0.889	0.001
Past MI	404 (15)	24 (19.4)	36 (27.5)	0.2	<0.001	0.141	<0.001
Hyperlipidemia	1315 (48.9)	70 (56.5)	77 (58.8)	0.12	0.031	0.8	0.027
Hypertension	1149 (42.8)	81 (65.3)	103 (78.6)	<0.001	<0.001	0.025	<0.001
Diabetes	617 (23)	41 (33.1)	50 (38.2)	0.012	<0.001	0.434	<0.001
Family history of IHD	616 (23)	19 (15.3)	5 (3.8)	0.048	<0.001	0.002	<0.001
Smoker	1365 (51.3)	50 (41)	38 (29.2)	0.026	<0.001	0.064	<0.001
Time to ER	120 [60–311]	200 [82–720]	180 [60–885]	<0.001	0.002	0.593	<0.001
Door to balloon	45 [30–60]	45 [30–60]	45 [30–60]	0.227	0.483	0.674	0.39
Time to reperfusion, min	175 [105–445]	270 [120–765]	240 [128–1060]	<0.001	0.002	0.861	<0.001
Coronary artery vessel disease				0.006	<0.001	0.451	<0.001
1	1130 (42.3)	43 (34.7)	36 (28.8)				
2	828 (31)	31 (25)	29 (23.2)				
3	700 (26.2)	50 (40.3)	60 (48)				
eGFR	78.42 ± 23.7	66.43 ± 24.4	50.53 ± 22.5	<0.001	<0.001	<0.001	<0.001
Creatinine admission	1.04 [0.9–1.19]	1.11 [0.97–1.29]	1.32 [1.06–1.71]	<0.001	<0.001	<0.001	<0.001
Creatinine peak	1.03 [0.92–1.18]	1.55 [1.38–1.98]	2.12 [1.65–2.96]	<0.001	<0.001	<0.001	<0.001
Troponin-I admission	0.91 [0.06–27.1]	0.73 [0.05–16.3]	2.95 [0.53–32.76]	0.457	0.002	0.003	0.005
Troponin-I peak	43.96 [6.92–384.95]	33.54 [2.65–126.25]	63.59 [7.35–348.79]	0.059	0.371	0.039	0.102

Note: Values for continuous variables are expressed as mean ± SD. Categorical Variables are expressed as number and percentage. Abbreviations: AKI, acute kidney injury; MI, myocardial infarction; IHD, ischemic heart disease; eGFR, estimated glomerular filtration rate; ER, emergency room.

**Table 2:** Logistic binary regression for no recovery/delayed recovery AKI adjusted for baseline variables found to be significant.

	HR	95% CI		*p*-Value
		Lower	Upper	
Age	1.063	1.044	1.082	<0.001
Family history of IHD	0.327	0.125	0.853	0.022
HTN	2.827	1.733	4.612	<0.001
CAD	1.304	1.031	1.650	0.027
EF	0.913	0.891	0.935	<0.001

Abbreviations: HR, hazard ratio; CI, Confidence interval; HTN, hypertension; IHD, ischemic heart disease; CAD, Coronary artery disease; EF, ejection fraction.

**Table 3:** Cox regression model for 1-year mortality.

	HR	95% CI		*p*-Value
		Lower	Upper	
Age	1.071	1.050	1.093	<0.001
No AKI	Reference			
AKI early recovery	2.091	0.933	4.687	0.073
AKI no recovery/delayed recovery	7.763	4.686	12.859	<0.001

Abbreviations: HR, hazard ratio; CI, Confidence interval; AKI, acute kidney injury.

## Data Availability

The data presented in this study are available on request from the corresponding author.

## Supplementary Materials

The following are available online at https://www.mdpi.com/article/10.3390/jcm11082169/s1, Table S1: In-hospital adverse outcomes.
