# Supplementary material for: Acute Kidney Injury Recovery Patterns in ST-Segment Elevation Myocardial Infarction Patients
Source: J Clin Med. 2022 Apr 13;11(8):2169. doi: 10.3390/jcm11082169 (PMC9025742; doi:10.3390/jcm11082169)
Supplement: Supplementary file 1 [file jcm-11-02169-s001.zip › jcm-1663281-supplementary.pdf]

Supplementary

Table S1. In-hospital adverse outcomes.

	**No AKI *n* = 2688**	**AKI Early Recovery *n* = 124**	**AKI no recovery *n* = 131**	***p* Value for No AKI vs AKI Early Recovery**	***p* Value for No AKI vs. AKI No Recover/Delayed Recovery**	***p* Value for AKI Early Recovery vs. AKI co Recovery/Delayed Recovery**	***p*-Value**
IABP/inotropes	72 (2.7)	16 (12.9)	38 (29)	<0.001	<0.001	0.002	<0.001
In hospital CABG	46 (1.7)	6 (4.8)	10 (7.6)	0.025	<0.001	0.443	<0.001
Mechanical ventilation	83 (3.1)	26 (21)	37 (28.2)	<0.001	<0.001	0.193	<0.001
VT/VF	185 (6.9)	22 (17.7)	20 (15.3)	<0.001	<0.001	0.616	<0.001
AF	104 (3.9)	16 (13)	24 (18.3)	<0.001	<0.001	0.302	<0.001
Stent thrombosis	104 (3.9)	9 (7.3)	7 (5.3)	0.094	0.357	0.61	0.135
Bleeding	122 (4.5)	21 (16.9)	22 (16.8)	<0.001	<0.001	1	<0.001
In hospital mortality	45 (1.7)	6 (5.1)	34 (26.4)	0.019	<0.001	<0.001	<0.001

Abbreviations: AKI, acute kidney injury; IABP, intra-aortic balloon pump; CABG, Coronary artery bypass graft surgery; VT, ventricular tachycardia; VF, ventricular fibrillation; AF, atrial fibrillation.
